# MR-proADM as Prognostic Factor of Outcome in COVID-19 Patients

**DOI:** 10.3390/biomedicines11061680

**Published:** 2023-06-09

**Authors:** Paolo Cameli, Elena Pordon, Miriana d’Alessandro, Maria Laura Marzi, Lucrezia Galasso, Cesare Biuzzi, Laura Bergantini, Elena Bargagli, Sabino Scolletta, Federico Franchi

**Affiliations:** 1 Respiratory Diseases Unit, Department of Medicine, Surgery and Neurosciences, University of Siena, 53100 Siena, Italy; paolocameli88@gmail.com (P.C.); e.pordon@student.unisi.it (E.P.); laurabergantini@gmail.com (L.B.); bargagli2@gmail.com (E.B.); 2 Anesthesia and Intensive Care Unit, Department of Medicine, Surgery and Neurosciences, University Hospital of Siena, 53100 Siena, Italy; marialaura.marzi@gmail.com (M.L.M.); cesare1205@gmail.com (C.B.); sabino.scolletta@dbm.unisi.it (S.S.); 3 Clinical Pathology Unit, Innovation, Experimentation and Clinical and Translational Research Department, University Hospital of Siena, 53100 Siena, Italy; lucrezia.galasso@dbm.unisi.it; 4 Cardiothoracic and Vascular Anesthesia and Intensive Care Unit, Department of Medical Science, Surgery and Neurosciences, University of Siena, 53100 Siena, Italy; federico.franchi@unisi.it

**Keywords:** COVID-19, KL-6, biomarkers, proADM

## Abstract

Background: Serum mid-regional proadrenomedullin (MR-proADM) has emerged as a marker of organ failure (mainly lungs and kidneys) and poor prognosis in patients admitted to intensive care (IC); some reports also suggest it and other markers, such as Krebs von den Lungen-6 (KL-6) and interleukin-6 (IL-6), as a prognostic biomarker of COVID-19. The aim of the study was to evaluate the performance MR-proADM in hospitalized COVID-19 patients for predicting in-hospital mortality and need for non-invasive or invasive respiratory support. Methods: We enrolled 74 patients hospitalized in the COVID Unit of Siena Hospital from March to May 2020, for whom serum samples were available on admission for assay of MR-proADM, KL-6 and IL-6. Demographic data, comorbidities, medical history and clinical laboratory data on days 1–3 of admission and Simplified Acute Physiology Score and Simplified Organ Failure Assessment scores calculated at day 1 were collected retrospectively, as well as mortality and IC admission data. Results: 12 patients died in hospital (16%) and 14 patients were admitted to IC (19%). Serum concentrations of MR-proADM on admission and on day 1 were higher among non-survivors than among survivors (*p* = 0.015 and *p* = 0.045, respectively), while those on day 3 were not significantly different. Patients needing respiratory support had higher MR-proADM concentrations on admission than the others (*p* = 0.046), and those requiring invasive mechanical ventilation had higher MR-proADM on day 1 (*p* = 0.017). Serum concentrations of KL-6 and IL-6 were significantly higher in non-survivors (*p* = 0.03 and *p* = 0.004, respectively). ROC curve analysis showed that serum MR-proADM on day 1 had the best accuracy in predicting death and/or IC admission (AUC = 0.9583, *p* = 0.0006); the combination of all three biomarkers further improved the accuracy of prediction of death or IC admission (AUC = 0.9793; *p* = 0.00004). Conclusions: Our data sustain the potential of serum MR-proADM as a reliable prognostic biomarker of hospitalized COVID-19 patients and confirms the utility of the three markers in the management and risk stratification of hospitalized patients. The markers are collected mini-invasively and are quick to analyze and cost-effective.

## 1. Background

The clinical spectrum of Coronavirus Disease 2019 (COVID-19) is variable, ranging from asymptomatic or mild symptoms to severe pneumonia, respiratory failure and multiorgan failure [1,2]. Mortality is higher in patients admitted to intensive care (IC), increasing from 15–20% in hospitalized patients to almost 40% in IC patients [3]. Although mass vaccination and approval of targeted drugs have reduced its mortality and morbidity [4], COVID-19 remains a real threat for elderly and frail patients, still being associated with a significant number of hospitalizations and deaths. The identification and validation of biomarkers that could help clinicians with early accurate severity assessment and prognosis is, therefore, still needed for correct triage, rational resource allocation and early identification of patients at risk of deterioration.

The pathogenesis of acute COVID-19 has been linked to a dysregulated immune response and septic hyperinflammation [5,6]. Biomarkers studied in such a setting could also be evaluated in the context of coronavirus infection [7,8]. Among these biomarkers, serum mid-regional proadrenomedullin (MR-proADM) emerges as a promising marker of organ failure, disease progression and poor prognosis. MR-proADM is a peptide derived 1:1 with ADM from a common precursor and, therefore, mirrors serum levels of ADM, which is produced by endothelial cells and has vasodilatory, natriuretic, diuretic, anti-inflammatory and immunomodulatory functions [9,10]. ADM is produced by endothelial cells and is ubiquitous in the human body; it exerts its activity by binding specific receptors in the lungs, kidneys, cardiovascular, gastrointestinal and endocrine systems. ADM is difficult to detect in blood because of its molecular instability and brief half-life. By contrast, MR-proADM has a half-life of hours, is easily quantified in plasma and has a reference range value of 0.26–0.51 nmoL/L. By virtue of these features, it has already been validated in clinical practice [11]. High levels of MR-proADM have been documented in septic patients in the first 24 h after diagnosis and have been linked to higher mortality. Its fast clearance under IC is predictive of better outcomes [12,13]. Serum MR-proADM has emerged as a marker of organ failure, particularly acute respiratory failure and acute kidney injury (AKI), and poor prognosis in an IC setting [13]. In patients admitted with respiratory failure, MR-proADM has shown better accuracy in predicting prognosis and 28-day outcome than other biomarkers such as procalcitonin, C-reactive protein, troponin and pro-brain natriuretic peptide [14]. Interestingly, it was recently proposed as a prognostic biomarker of COVID-19 in hospitalized patients [15,16]. COVID-19 is associated with various degrees and types of endothelial dysfunction [17]. Many histopathological studies have demonstrated a direct viral cytopathic effect associated with significant infiltration of inflammatory cells in endothelium, further suggesting that endotheliitis is directly related to disease severity [18,19]. Faced with the need for reliable and cost-effective biomarkers for COVID-19 patients to optimize management and prognostic estimation, MR-proADM may be useful in clinical practice for detecting and/or predicting patients most at risk of developing organ failure.

In this field, another proinflammatory cytokine and oxidative stress mediator of lung injury is Krebs von den Lungen-6 (KL-6), a mucin protein produced mainly by damaged or regenerating alveolar type II pneumocytes. It is recognized as a prognostic bioindicator of interstitial lung diseases [20], especially idiopathic pulmonary fibrosis and progressive pulmonary fibrosis [21], and it may also be associated with response to antifibrotic treatment [22,23]. Since early 2020, KL-6 has showed promise as a prognostic marker of COVID-19, although most studies have been single-center and with small numbers of patients [24,25].

Since the beginning of the COVID-19 pandemic, serum IL-6 has emerged as a predictor of severity and prognosis in hospitalized patients, probably due to its crucial role in triggering the ‘cytokine storm’ leading to acute disease. This assumption is confirmed by evidence from clinical trials investigating the efficacy of anti-IL-6 drugs in patients with severe COVID-19 [26].

Since COVID-19 is still a global threat to health systems due to the associated morbidity, hospitalization risk and mortality, the aim of the present study was to investigate the performance of serum MR-proADM, alone and combined with KL-6 and IL-6, in predicting in-hospital mortality and the need for invasive respiratory support on admission to hospital.

## 2. Methods

This monocentric, prospective, observational study was conducted at Siena University Hospital. All adult patients (age over 18 years) hospitalized for SARS-CoV-2 infection and admitted to the COVID Unit between March and May 2020 were enrolled prospectively. Inclusion criteria included informed consent to serum sampling and storage, and the blood sampling for biomarker assessment had to have been performed at admission and on days 1 and 3 after admission. Data were collected during the hospitalization period until discharge or death, whichever came first. All patients included in the study underwent a nasopharyngeal swab for SARS-CoV-2 infection on admission. The following data were recorded: age, gender, comorbidities, previous therapies, date of onset and nature of symptoms, clinical presentation, antiviral and immunosuppressive treatments, need for respiratory or organ support, length of stay, complications and in-hospital death. The following laboratory results were recorded on admission, day 1 and day 3: complete blood count, C-reactive protein (CRP), procalcitonin, ferritin, D-Dimer, prothrombin time (PT), international normalized ratio (INR), activated partial thromboplastin time (aPTT), aspartate aminotransferase, alanine aminotransferase, albumin, bilirubin, creatinine, LDH, pro-BNP and arterial blood gas parameters. The same timeline for blood sampling was used for MR-proADM assessment. SAPS II (Simplified Acute Physiology Score) and SOFA (Simplified Organ Failure Assessment) scores were calculated at day 1. All data were entered in an anonymous electronic database.

Patients with known interstitial lung disease (ILD), chronic obstructive lung disease, lung or pleural cancer or concomitant infections were excluded from the study, as well as patients under 18 years or unable to give informed consent. Patients who died or were discharged in the first 3 days of hospitalization and were, therefore, unable to perform all blood samplings were excluded from the analysis. If a patient was admitted to IC in the first 3 days of hospitalization, only pre-IC blood samples were used for biomarker assessment and subsequently included in the statistical analysis.

According to our COVID Unit protocol, hospitalized patients were only admitted to IC when orotracheal intubation due to respiratory failure was prescribed; patients needing high-flow oxygen therapy and/or non-invasive ventilation were allocated to sub-intensive care and managed by a multidisciplinary team, including respiratory physicians, cardiologists and intensive care physicians.

In a subgroup of 22 patients, serum sampling for KL-6 and IL-6 assay performed on the same day as baseline MR-proADM was available and biomarker levels were included for outcome analysis.

The study was conducted according to the principles of the Declaration of Helsinki and was approved by the local Ethical Committee (code BIOCOVID, C.E.A.V.S.E. protocol number 180409).

### 2.1. Serum Biomarker Assay

Blood and serum samples were collected on admission and on days 1 and 3 of hospital stay. Preparation and storage of serum was performed at the Clinical Pathology Laboratory, University Hospital of Siena. Peripheral blood samples were collected in a tube containing ethylenediamine tetra acetic acid (EDTA) anticoagulant (BD Vacutainer^®^ EDTA tubes; BD Biosciences, San Jose, CA, USA) and centrifuged (15,000× *g* for 10 min); plasma was stored at −80 °C until assay. The method described by Sozio et al. [16] was used to measure plasma concentrations of MR-proADM in our population (automated Kryptor analyzer, time-resolved amplified cryptate emission (TRACE) technology assay (Kryptor PCT; Brahms AG; Hennigsdorf, Germany) and commercially available immunoluminometric assay kits (Brahms)). Serum concentrations of KL-6 (sKL-6) were measured by KL-6 reagent assay (Fujirebio Europe, Gent, Belgium). The principle of the assay is agglutination of sialylated carbohydrate antigen in samples with KL-6 monoclonal antibody by antigen–antibody reaction. The change in absorbance was measured to determine KL-6 concentrations, which were expressed in U/mL.

### 2.2. Statistical Analysis

Continuous and categorical variables were reported as median [interquartile range, IQR] and n (%), respectively. The Kolmogorov–Smirnov test was used to assess distribution of variables. Considering mortality and/or IC admission as the main outcomes and the predicted incidence of mortality for hospitalized COVID-19 patients in the Wuhan population [1], we calculated that we needed to enroll a total of 80 subjects in the study, since a total sample size of 70 patients would have a minimum power of 80% to detect differences and similarities in primary variables. Differences between continuous variables were compared using the Student’s *t*-test or Mann–Whitney test, while differences between categorical variables were compared with Fisher’s exact or chi-squared tests, as appropriate. The predictive value of MR-proADM for the predefined outcomes was assessed by area under the receiver operating characteristic curve (AUROC). Sensitivity, specificity and positive and negative predictive values (PPV and NPV, respectively,) were calculated for MR-proADM. The Youden index (J = max [sensitivity + specificity − 1]) was used to establish the best cut-off. The multivariate Cox proportional risk model was used to define independent variables related to in-hospital death and development of complications. Odds ratios (ORs) were computed with a 95% confidence interval. A *p*-value < 0.05 was considered statistically significant. Statistical analysis and figure generation were conducted with GraphPad Prism software (version 5) and SPSS Statistics (version 29.0).

## 3. Results

### 3.1. Study Population

**Figure 1:**
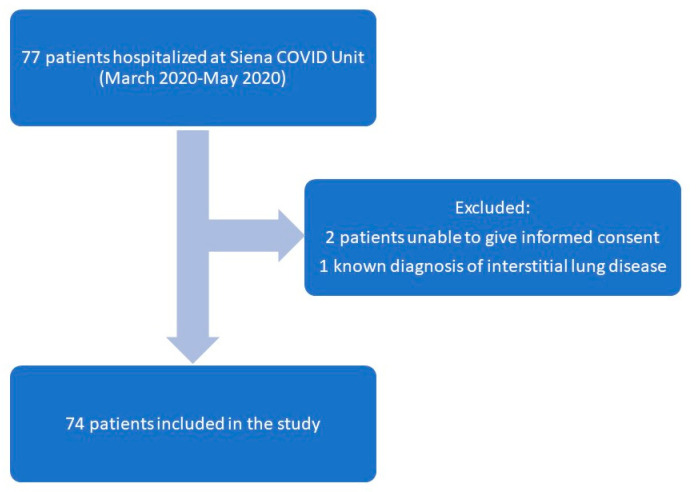
Flow diagram describing the enrollment of study population. Blood samplings at admission and day 1 of hospital stay were available for all patients included in the study. Two patients were admitted to ICU by day 3.

From March to May 2020, 77 patients were hospitalized in the Siena COVID Unit after testing positive to nasopharyngeal swabs performed in the previous 24 h. Applying the exclusion criteria, we prospectively recruited 74 patients (49 males, 67.9 ± 15.4 years old). The three patients excluded were not eligible because two were unconscious on admission and one reported a previous diagnosis of ILD (Figure 1). Two patients were admitted to IC on day 2, and in line with the study protocol, blood samples for MR-proADM assessment were not obtained on day 3.

Table 1 shows the main data of the study population. Common clinical presentations in descending order were fever (74%), coryza (66%), dyspnea (55%), cough (47%), gastrointestinal symptoms (24%) and chest pain (5%). Comorbidities included hypertension (58%), diabetes mellitus (20%), hypothyroidism (15%), dyslipidemia (12%), chronic atrial fibrillation (12%) and chronic obstructive pulmonary disease (COPD) (12%) without any differences between survivors and non-survivors.

### 3.2. Survival, IC and Outcomes

In the study population, 12 patients died in hospital (16%) and 14 patients were admitted to IC (19%). Mortality in IC was 29%. Non-survivors were significantly older than survivors (86 [71.5–89] years vs. 65 [55–75.2] years, *p* < 0.0001) while gender and smoking differences were not significant.

During hospital stay, the following complications developed: bacterial super-infection (28%), AKI (17%), pleural effusion (16%), atrial fibrillation (12%), gastrointestinal disease (10%), liver failure (4%) and pneumothorax (4%). AKI and pleural effusions were significantly more frequent in non-survivors than in survivors (41.7% vs. 6.7%, *p* = 0.005 and 50% vs. 9.7%, *p* = 0.001, respectively), and patients developing AKI were significantly older (79 [69–86] years vs. 67 [56–79] years, *p* = 0.045).

A total of 55 patients (74%) needed conventional oxygen therapy (COT) and 21 (28%) needed at least one type of respiratory support (27 cases altogether). HFNC was employed in 10 cases (37%), continuous positive airway pressure (CPAP) with helmet in 20 (74%), non-invasive ventilation (NIV) in 1 (3.7%) and invasive mechanical ventilation (IMV) in 14 (52%). Half the patients who underwent invasive ventilation were placed in prone position and tracheostomy was performed in 5 cases (Table 2). Overall mortality in patients with respiratory support of any type was significantly higher than in patients without it or on COT (75% vs. 25%, *p* = 0.006).

### 3.3. Biomarker Assessment

No significant differences in demographic features, clinical outcome and laboratory values were observed between the entire study population and the subgroup of 22 patients for whom all MR-proADM, KL-6 and IL-6 values were available (Table S1). Concerning the principal outcome of the study (death and/or IC), no significant difference was observed between these two subgroups (14/52 vs. 8/22, *p* = 0.4202).

**Figure 2:**
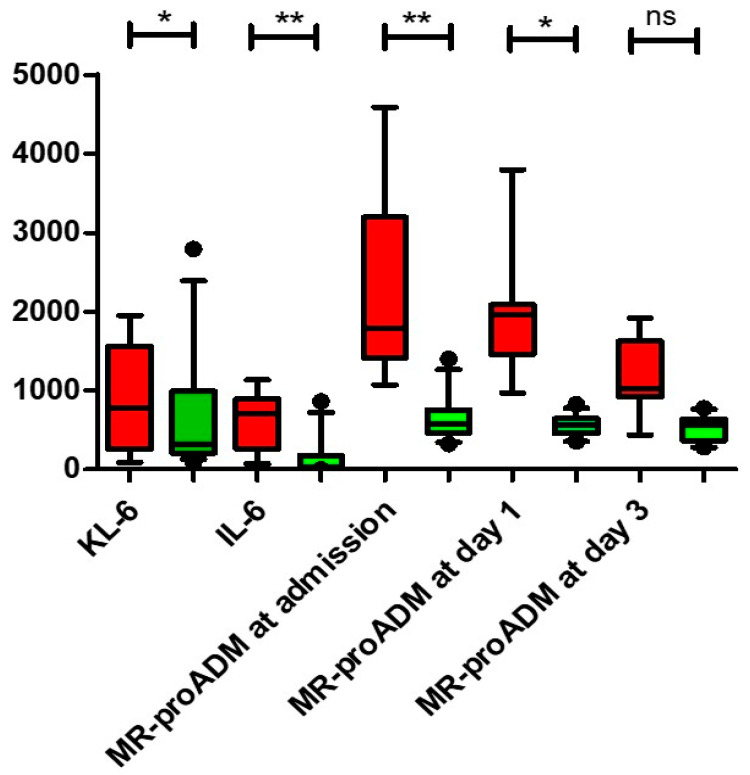
Comparison of serum biomarkers’ concentrations between survivors (green box) and non-survivors (red box). All serum biomarkers, with the exception of MR-proADM at day 3, were significantly higher in patients with a negative outcome than in patients who were discharged. KL-6 and IL-6 levels were measured at hospital admission. Data are expressed as median ± interquartile range. MR-proADM, KL-6 and IL-6 levels are expressed as µmoL/L, U/mL and pg/mL, respectively. *: *p* < 0.05; **: *p* < 0.001. Abbreviations: ns, not significant; KL-6, Krebs von den Lungen-6; IL-6, interleukin-6.

MR-proADM levels on admission and on day 1 were higher among non-survivors than among survivors (2.31 [1.25–3.9] nmol/L vs. 0.74 [0.53–1.02] nmol/L, *p* = 0.001 and 1.82 [1.12–5.25] nmol/L vs. 0.69 [0.53–1.07] nmol/L, *p* = 0.045, respectively), while levels on day 3 were not significantly different (Figure 2).

Patients needing respiratory support (HFNC and/or cPAP) had higher MR-proADM concentrations on admission than the others (1.1 [0.8–2.2] nmoL/L vs. 0.7 [0.5–1.0] nmoL/L, *p* = 0.046). Those requiring invasive mechanical ventilation (IMV) had higher MR-proADM levels on day 1 (1.8 [0.7–2.9] nmoL/L vs. 1.1 [0.7–1.0], *p* = 0.017). Patients who developed AKI also showed higher MR-proADM levels on admission (2.5 [1,2,3,4] nmoL/L vs. 1.1 [0.8–1.3] nmoL/L, *p* = 0.001).

Serum concentrations of KL-6 and IL-6 were significantly higher in non-survivors (875 [403–1678] vs. 306 [158–1405] U/mL, *p* = 0.03 and 769 [285.5–906.4] vs. 38.6 [7–167.4] pg/mL, *p* = 0.004, respectively) (Figure 1); the same was found when death and/or IC were taken as the main outcome (903 [437–1773] vs. 317 [212–1396] U/mL, *p* = 0.04 and 701 [263.5–895.3] vs. 35.1 [7.4–178.2] pg/mL, *p* = 0.004, respectively), while no differences were observed between those who did or did not develop AKI (*p* = 0.525 and *p* = 0.654, respectively).

Concerning other serum tests commonly used in clinical practice, we observed that only CRP at admission was significantly higher in non-survivors (8.6 [4.8–18.6] vs. 4.8 [4–15.8] mg/dL, *p* = 0.03); similar results were obtained taking death and/or IC as the main outcome (8.1 [1.8–32.7] vs. 4.6 [4.1–8.9], *p* = 0.02).

### 3.4. ROC Curve Analysis

ROC curve analysis showed that MR-proADM at baseline and on day 1 had the best accuracy in predicting death and/or IC (AUC = 0.8269, *p* = 0.013 and AUC = 0.9583, *p* = 0.0006, respectively), as well as severe respiratory failure (needing cPAP and/or HFNC) and/or IC with orotracheal intubation (AUC = 0.8056, *p* = 0.019 and AUC = 0.9091, *p* = 0.0021, respectively). IL-6 also showed good accuracy for death or IC (AUC = 0.8796, *p* = 0.0035), while KL-6 did not reach statistical significance (AUC = 0.6731, *p* = 0.193). CRP at admission also showed fair accuracy in predicting all-cause mortality or death and/or IC in our study population (AUC = 0.6653, *p* = 0.0346 and AUC = 0.6875, *p* = 0.0262), albeit with lower significance than MR-proADM on day 1 (difference in AUC: 0.293, *p* = 0.000067) and IL-6 (difference in AUC: 0.2143, *p* = 0.0146).

**Figure 3:**
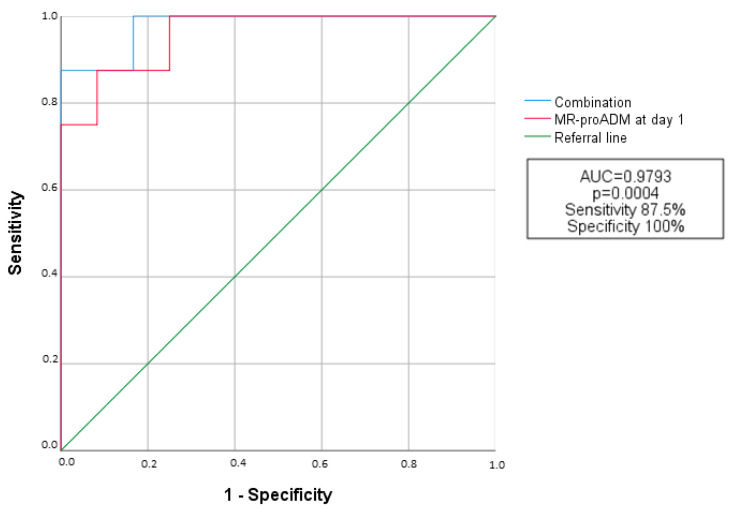
Comparison of ROC curve analysis curves between MR-proADM at day 1 and the combination of MR-proADM at day 1 plus KL-6 at admission and IL-6 at admission for predictive accuracy of death or ICU admission. The combination of three biomarkers shows a higher accuracy in predicting a negative outcome than MR-proADM at day 1 alone, though not significantly (difference of AUC = 0.021, *p* = 0.7021). MR-proADM: Mid-Regional proadrenomedullin.

When we combined all three biomarkers (MR-proADM on day 1, KL-6 and IL-6), we observed a further improvement in accuracy in predicting death or IC (AUC = 0.9793; *p* = 0.00004) (Figure 3), that was statistically significant compared to KL-6 (difference in AUC = 0.3062, *p* = 0.00766) and IL-6 (difference in AUC = 0.0997; *p* = 0.0116), but not compared to MR-proADM on day 1 (difference in AUC = 0.021, *p* = 0.7021).

### 3.5. Univariate and Multivariate Analysis

Univariate analysis showed that patients with MR-proADM ≥ 1.02 nmoL/L at admission were significantly older (82 [71–87] year vs. 63 [55–73] years, *p* < 0.000), more often had arterial hypertension (81.8% vs. 47.7%, *p* = 0.009), chronic atrial fibrillation (27.3% vs. 4.5%, *p* = 0.014) and chronic renal failure (18.2% vs. 0%, *p* = 0.01) and were likely to develop AKI (30.4% vs. 2.2%, *p* = 0.002), bacterial super-infections (45.5% vs. 17.8%, *p* = 0.022) and pleural effusions (39.1% vs. 4.3%, *p* = 0.003). Moreover, respiratory support was more frequently needed (61.9% vs. 25.6%, *p* = 0.007), and patients were likely to be intubated (ns). Minimum PaO_2_/FiO_2_ was lower in this group of patients (1.5 [0.7–3.6] vs. 2.8 [2–3.5], *p* = 0.002). Mortality was higher in patients with MR-proADM ≥ 1.02 nmoL/L at admission (39.1% vs. 2.2%, *p* = 0.019).

In the multivariate analysis with mortality as the dependent variable, only age, MR-proADM level at admission and the combination of MR-proADM, KL-6 and IL-6 (all at admission) proved to be independent predictors of this outcome. MR-proADM and LDH at admission predicted need for respiratory support in hospital, while only the combination of biomarkers (MR-proADM + KL-6 + IL-6 at admission) was found to be associated with IC and IMV. With AKI as the dependent variable, only MR-proADM at baseline predicted this complication (Table 3).

## 4. Discussion

Recent studies have suggested serum MR-proADM as a potential prognostic biomarker of COVID-19 [15,27]. We confirmed these results in a monocentric prospective observational study conducted at Siena University Hospital in which the prognostic value of MR-proADM was compared for the first time with other COVID-19 markers of lung damage, including KL-6 and IL-6. The potential utility of a combination of these biomarkers in clinical practice was also evaluated.

Considering the resource allocation issues associated with the COVID-19 pandemic, a biomarker that can predict patients at high risk of severe disease is surely of primary interest. In our cohort, patients who developed more severe forms and had higher mortality were older, more often needed respiratory support and developed complications such as AKI and pleural effusions; these findings were consistent with the literature [1,28]. In this population, MR-proADM levels > 1.02 nmoL/L at admission were associated with a higher risk of mortality, respiratory failure (indicated by need for any type of respiratory device) and AKI as a complication. Our findings substantially confirmed the results published by Sozio et al. [14], who showed good accuracy of MR-proADM in predicting more severe cases and/or need for IC at the time of hospitalization. Our findings are further strengthened by the standardized prospective study design, which allowed us to standardize serum sampling, clinical features and outcome data collection, and by multivariate analysis, which shows the reliability of this biomarker for prognostic estimation. Hence, a single detection at admission could guide clinicians in the correct triage of patients, identifying those at risk of mortality and of developing severe forms of the disease. To our knowledge, this is the first study in which serum concentrations of MR-proADM were measured not only on admission to hospital but also on days 1 and 3 of hospital stay. We did not observe any significant trend in the study population as a whole, but a decreasing trend of this marker was found in the subgroup of patients with the worst outcome. These findings may be explained by evidence that endothelial dysfunction occurs in an early phase of COVID-19 disease, prior to onset of ARDS or multiorgan failure [17,18]. This hypothesis is also supported by previous reports investigating the role of MR-proADM in critical patients, where this marker proved to be promising in predicting but not in assessing the severity of organ failure [29,30].

Interestingly, our results suggest that MR-proADM at admission has better performance in predicting severe disease and mortality than CRP. This finding is of great interest, since serum CRP has been reported to be one of the most reliable prognostic indicators in large cohorts of hospitalized COVID-19 patients [2,31]. This issue was also addressed in the paper by Sozio et al., in which MR-proADM still showed a higher AUC than CRP in predicting need for IC (particularly for patients < 64 years of age) and in which the authors proposed a conditional decision tree for patient allocation according to the combination of these two biomarkers [15]. Though underpowered for a reliable confirmation of these findings, our results substantially confirmed a slightly better accuracy of MR-proADM than CRP in predicting a negative outcome in COVID-19 patients. This may be explained by its higher specificity for endothelial damage, which in turn is associated with the severest forms of the disease [17,18]. The potential of endothelial-derived serum proteins for a more accurate prognostic estimation in this field is further sustained by studies investigating the performance of other biomarkers (such as serum von Willebrand factor, endocan and vascular endothelial growth factor) in predicting mortality, need for IC or major thrombotic events [32,33,34,35]. Unfortunately, we did not include evaluation of these biomarkers in our study protocol, so we were unable to compare MR-proADM values with other markers of endothelial dysfunction.

We first proposed a combination of serum markers, all of which have already demonstrated their potential in previous studies [36]. Although values of all three biomarkers were only available for a minority of our study population, our results showed that the combination of MR-proADM, KL-6 and IL-6 improved accuracy for outcome prediction and may be valuable in the clinical management of hospitalized COVID-19 patients. This approach is sustained by evidence that COVID-19 is associated not only with a respiratory syndrome but also with multiple organ failure that may be fatal or require admission to IC for non-respiratory causes too. This assumption may also explain the better performance of MR-proADM and IL-6 than KL-6 observed in our study, considering the specificity of the latter for respiratory tissue damage. In this regard, our evidence of higher serum concentrations of MR-proADM (but not KL-6 or IL-6) in patients experiencing AKI while in hospital further confirms the potential of this biomarker for prognostic estimation of organ failure. Our findings are in line with the literature, since MR-proADM had already been reported to be a good predictive marker of AKI and need for renal replacement therapy in critical patients with or without COVID-19, probably due to its reliability as a marker of capillary leakage and endothelial dysfunction [29,37,38]. Since AKI is considered a major negative prognostic factor in hospitalized COVID-19 patients [39], these findings are important and are in line with previous reports focused on non-COVID-19 patients in IC [13].

The present study has many limitations. First of all, the monocentric design and quite small study population limit the scientific soundness of our findings, although the prospective design of patient inclusion prevents reporting and recalling bias. Moreover, since the study population was enrolled in the first phase of the COVID-19 pandemic, and COVID-19 syndrome has maintained quite similar clinical features in hospitalized patients since 2020, our findings may not be completely suitable for the subsequent SARS-CoV-2 variants. We have no data, therefore, on serum biomarker expression in vaccinated or re-infected patients, or in those treated with antivirals.

In conclusion, although our findings need confirmation in a larger cohort in order to be implemented in clinical practice, they are certainly promising; the three biomarkers included in our study are readily collected in a mini-invasive manner, quick to analyze and cost-effective. They promise, therefore, to be an intriguing new tool for more accurate risk stratification and early treatment.

## Figures and Tables

**Table 1:** Demographic features, clinical and treatment data and biomarker assessment of study population at admission, day 1 and day 3 of hospital stay.

Parameter	Total Population			
N	74			
Age (years)	67.9 ± 15.4			
Male gender (%)	44 (59%)			
Medical history				
- Arterial hypertension (%)	43 (58%)			
- Diabetes mellitus (%)	15 (20.2%)			
- Atrial fibrillation (%)	11 (14.8%)			
- Chronic kidney disease (%)	5 (6.7%)			
- Chronic obstructive pulmonary disease (%)	9 (12%)			
- Active/previous cancer (%)	6 (8.1%)			
	Admission	Day 1	Day 3	*p*-value
Clinical assessment				
- SOFA	n.a.	3.1 ± 2.4	n.a.	
- SAPS II	n.a.	26 ± 12.3	n.a.	
- Hemoglobin (g/dL)	13.1 ± 1.9	12.4 ± 1.9	12.6 ± 1.5	0.0621
- WBC (×10^3^/mmc)	7.4 ± 4.4	7.5 ± 4.2	7.3 ± 3.1	0.6773
- Lymphocytes (×10^3^/mmc)	1.07 ± 0.8	0.92 ± 0.53	1.04 ± 0.68	0.6911
- Neutrophils (×10^3^/mmc)	5.61 ± 4.22	6.05 ± 3.91	5.59 ± 2.97	0.4849
- Platelets (×10^3^/mmc)	217.3 ± 92.7	228.4 ± 104.5	261.7 ± 112.3	0.0155 *
- Creatinine (mg/dL)	1.2 ± 0.9	1.15 ± 0.9	0.9 ± 0.7	0.1919
- CRP (mg/dL)	9.9 ± 20.3	8.6 ± 8.2	6.2 ± 7.4	0.1438
- PCT (ng/mL)	0.8 ± 2.6	1.6 ± 4.3	0.3 ± 0.3	0.5669
- INR	1.3 ± 0.9	1.2 ± 0.4	1.2 ± 0.1	0.8612
- D-Dimer (FEU/mL)	1279 ± 1070	2043 ± 3751	8781 ± 27,965	0.4987
- Bilirubin (mg/dL)	0.8 ± 2.6	1.03 ± 3.4	1.1 ±4.2	0.3242
- LDH (U/L)	321 ± 133	340 ± 146	297 ± 115	0.4942
- pro-BNP (pg/mL)	723 ± 1225			
- pH	6.9 ± 1.7			
- paCO_2_ (mmHg)	33.6 ± 11.1			
- paO_2_ (mmHg)	81.1 ± 33.4			
- paO_2_/FiO_2_ (mmHg/%)	2.9 ± 1.2			
- Serum lactate (mmol/L)	1.3 ± 0.4			
Biomarker analysis				
- MR-proADM (nmoL/L)	1.2 ± 1.3	1.2 ± 1.3	1.1 ± 1 **	0.7772
- KL-6 (U/mL)	1279 ± 2484			
- IL-6 (pg/mL)	26 ± 36.7			

Data are reported as mean ± standard deviation. SOFA: sequential organ failure assessment; SAPS: Simplified Acute Physiology Score; WBC: white blood cells; CRP: C-reactive protein; PCT: procalcitonin; INR: international normalized ratio; LDH: lactate dehydrogenase; pro-BNP: N-terminal pro-brain natriuretic peptide; MR-proADM: mid-regional proadrenomedullin; KL-6: Krebs von den Lungen-6; IL-6: interleukin-6. *: statistical significance between admission and day 3 by Dunn’s post test; **: MR-proADM was available for 72 patients (the exceptions were two patients admitted to IC between days 1 and 3).

**Table 2:** Comparison of respiratory support data of survivors and non-survivors. COT, conventional oxygen therapy; HFNC, high-flow nasal cannula; CPAP, continuous positive airway pressure; NIMV, non-invasive mechanical ventilation; IMV, invasive mechanical ventilation; ns: not significant.

	All (n = 74)	Survivors (n = 62)	Non-Survivors (n = 12)	*p*-Value
COT, n (%)	55 (74.3)	47 (75.8)	8 (66.7)	ns
HFNC, n (%)	10 (13)	7 (11.3)	3 (25)	ns
CPAP, n (%)	20 (27)	15 (24.2)	5 (41.7)	ns
NIMV, n (%)	1 (1.4)	-	1 (8)	ns
IMV, n (%)	14 (18.9)	10 (16.1%)	4 (33.3)	ns
Tracheostomy	5 (6.8)	3 (4.8)	2 (16.7)	ns

**Table 3:** Multivariate analysis for outcomes.

	OR	95% C.I.	*p*-Value
Mortality			
MR-proADM + KL-6 + IL-6	2.506	1.786–3.568	0.003
MR-proADM at admission	1.816	1.135–2.906	0.013
Age	1.080	1.005–1.160	0.036
Respiratory support			
MR-proADM + KL-6 + IL-6	1.567	0.885–2.256	0.056
MR-proADM at admission	1.789	1.067–2.999	0.027
LDH at admission	1.010	1.004–1.017	0.002
ICU admission			
MR-proADM + KL-6 + IL-6	1.746	1.125–3.054	0.016
LDH at admission	1.005	1.000–1.010	0.063
AKI			
MR-proADM at admission	1.824	1.155–2.882	0.010

## Data Availability

All data generated or analyzed during the study is included in this published article.

## Supplementary Materials

The following supporting information can be downloaded at: https://www.mdpi.com/article/10.3390/biomedicines11061680/s1, Table S1. Baseline demographic features, clinical and laboratory data of study population, stratified according to the availability of serum biomarkers.

## References

[1] Zhu N., Zhang D., Wang W., Li X., Yang B., Song J., Zhao X., Huang B., Shi W., Lu R. (2020). A Novel Coronavirus from Patients with Pneumonia in China, 2019. N. Engl. J. Med..

[2] Ponti G., Maccaferri M., Ruini C., Tomasi A., Ozben T. (2020). Biomarkers Associated with COVID-19 Disease Progression. Crit. Rev. Clin. Lab. Sci..

[3] Wiersinga W.J., Rhodes A., Cheng A.C., Peacock S.J., Prescott H.C. (2020). Pathophysiology, Transmission, Diagnosis, and Treatment of Coronavirus Disease 2019 (COVID-19): A Review. JAMA.

[4] Havers F.P., Pham H., Taylor C.A., Whitaker M., Patel K., Anglin O., Kambhampati A.K., Milucky J., Zell E., Moline H.L. (2022). COVID-19-Associated Hospitalizations Among Vaccinated and Unvaccinated Adults 18 Years or Older in 13 US States, January 2021 to April 2022. JAMA Intern. Med..

[5] Giamarellos-Bourboulis E.J., Netea M.G., Rovina N., Akinosoglou K., Antoniadou A., Antonakos N., Damoraki G., Gkavogianni T., Adami M.-E., Katsaounou P. (2020). Complex Immune Dysregulation in COVID-19 Patients with Severe Respiratory Failure. Cell Host Microbe.

[6] Noroozi R., Branicki W., Pyrc K., Łabaj P.P., Pospiech E., Taheri M., Ghafouri-Fard S. (2020). Altered Cytokine Levels and Immune Responses in Patients with SARS-CoV-2 Infection and Related Conditions. Cytokine.

[7] (2022). COVID-19 Host Genetics Initiative A First Update on Mapping the Human Genetic Architecture of COVID-19. Nature.

[8] Daga S., Fallerini C., Baldassarri M., Fava F., Valentino F., Doddato G., Benetti E., Furini S., Giliberti A., Tita R. (2021). Employing a Systematic Approach to Biobanking and Analyzing Clinical and Genetic Data for Advancing COVID-19 Research. Eur. J. Hum. Genet..

[9] Carrizo G.J., Wu R., Cui X., Dwivedi A.J., Simms H.H., Wang P. (2007). Adrenomedullin and Adrenomedullin-Binding Protein-1 Downregulate Inflammatory Cytokines and Attenuate Tissue Injury after Gut Ischemia-Reperfusion. Surgery.

[10] Temmesfeld-Wollbrück B., Brell B., Dávid I., Dorenberg M., Adolphs J., Schmeck B., Suttorp N., Hippenstiel S. (2007). Adrenomedullin Reduces Vascular Hyperpermeability and Improves Survival in Rat Septic Shock. Intensive Care Med..

[11] Lorubbio M., Conti A.A., Ognibene A. (2018). Midregional Pro-Adrenomedullin (MR-ProADM) Reference Values in Serum. Clin. Biochem..

[12] Spoto S., Nobile E., Carnà E.P.R., Fogolari M., Caputo D., De Florio L., Valeriani E., Benvenuto D., Costantino S., Ciccozzi M. (2020). Best Diagnostic Accuracy of Sepsis Combining SIRS Criteria or QSOFA Score with Procalcitonin and Mid-Regional pro-Adrenomedullin Outside ICU. Sci. Rep..

[13] Elke G., Bloos F., Wilson D.C., Brunkhorst F.M., Briegel J., Reinhart K., Loeffler M., Kluge S., Nierhaus A., Jaschinski U. (2018). The Use of Mid-Regional Proadrenomedullin to Identify Disease Severity and Treatment Response to Sepsis—A Secondary Analysis of a Large Randomised Controlled Trial. Crit. Care Lond. Engl..

[14] Choi J.J., McCarthy M.W. (2018). The Prognostic Value of Mid-Regional pro-Adrenomedullin in the Evaluation of Acute Dyspnea. Expert Rev. Mol. Diagn..

[15] Sozio E., Moore N.A., Fabris M., Ripoli A., Rumbolo F., Minieri M., Boverio R., Rodríguez Mulero M.D., Lainez-Martinez S., Martínez Martínez M. (2022). Identification of COVID-19 Patients at Risk of Hospital Admission and Mortality: A European Multicentre Retrospective Analysis of Mid-Regional pro-Adrenomedullin. Respir. Res..

[16] Sozio E., Tascini C., Fabris M., D’Aurizio F., De Carlo C., Graziano E., Bassi F., Sbrana F., Ripoli A., Pagotto A. (2021). MR-ProADM as Prognostic Factor of Outcome in COVID-19 Patients. Sci. Rep..

[17] Pons S., Fodil S., Azoulay E., Zafrani L. (2020). The Vascular Endothelium: The Cornerstone of Organ Dysfunction in Severe SARS-CoV-2 Infection. Crit. Care Lond. Engl..

[18] Varga Z., Flammer A.J., Steiger P., Haberecker M., Andermatt R., Zinkernagel A.S., Mehra M.R., Schuepbach R.A., Ruschitzka F., Moch H. (2020). Endothelial Cell Infection and Endotheliitis in COVID-19. Lancet Lond. Engl..

[19] Ackermann M., Verleden S.E., Kuehnel M., Haverich A., Welte T., Laenger F., Vanstapel A., Werlein C., Stark H., Tzankov A. (2020). Pulmonary Vascular Endothelialitis, Thrombosis, and Angiogenesis in Covid-19. N. Engl. J. Med..

[20] d’Alessandro M., Bergantini L., Cameli P., Lanzarone N., Antonietta Mazzei M., Alonzi V., Sestini P., Bargagli E. (2020). Serum KL-6 Levels in Pulmonary Langerhans’ Cell Histiocytosis. Eur. J. Clin. Investig..

[21] d’Alessandro M., Bergantini L., Cameli P., Vietri L., Lanzarone N., Alonzi V., Pieroni M., Refini R.M., Sestini P., Bonella F. (2020). Krebs von Den Lungen-6 as a Biomarker for Disease Severity Assessment in Interstitial Lung Disease: A Comprehensive Review. Biomark. Med..

[22] d’Alessandro M., Bergantini L., Cameli P., Pieroni M., Refini R.M., Sestini P., Bargagli E. (2021). Serum Concentrations of KL-6 in Patients with IPF and Lung Cancer and Serial Measurements of KL-6 in IPF Patients Treated with Antifibrotic Therapy. Cancers.

[23] Nakano A., Ohkubo H., Fukumitsu K., Fukuda S., Kanemitsu Y., Takemura M., Maeno K., Ito Y., Oguri T., Niimi A. (2019). Remarkable Improvement in a Patient with Idiopathic Pulmonary Fibrosis after Treatment with Nintedanib. Intern. Med. Tokyo Jpn..

[24] d’Alessandro M., Cameli P., Refini R.M., Bergantini L., Alonzi V., Lanzarone N., Bennett D., Rana G.D., Montagnani F., Scolletta S. (2020). Serum KL-6 Concentrations as a Novel Biomarker of Severe COVID-19. J. Med. Virol..

[25] d’Alessandro M., Bergantini L., Cameli P., Curatola G., Remediani L., Bennett D., Bianchi F., Perillo F., Volterrani L., Mazzei M.A. (2021). Serial KL-6 Measurements in COVID-19 Patients. Intern. Emerg. Med..

[26] Rosas I.O., Bräu N., Waters M., Go R.C., Hunter B.D., Bhagani S., Skiest D., Aziz M.S., Cooper N., Douglas I.S. (2021). Tocilizumab in Hospitalized Patients with Severe Covid-19 Pneumonia. N. Engl. J. Med..

[27] Fialek B., De Roquetaillade C., Pruc M., Navolokina A., Chirico F., Ladny J.R., Peacock F.W., Szarpak L. (2023). Systematic Review with Meta-Analysis of Mid-Regional pro-Adrenomedullin (MR-Proadm) as a Prognostic Marker in Covid-19-Hospitalized Patients. Ann. Med..

[28] Yang X., Yu Y., Xu J., Shu H., Xia J., Liu H., Wu Y., Zhang L., Yu Z., Fang M. (2020). Clinical Course and Outcomes of Critically Ill Patients with SARS-CoV-2 Pneumonia in Wuhan, China: A Single-Centered, Retrospective, Observational Study. Lancet Respir. Med..

[29] Nierhaus A., Bloos F., Wilson D.C., Elke G., Meybohm P. (2018). SepNet Critical Care Trials Group Predicting the Requirement for Renal Replacement Therapy in Intensive Care Patients with Sepsis. Crit. Care Lond. Engl..

[30] Saeed K., Wilson D.C., Bloos F., Schuetz P., van der Does Y., Melander O., Hausfater P., Legramante J.M., Claessens Y.-E., Amin D. (2019). The Early Identification of Disease Progression in Patients with Suspected Infection Presenting to the Emergency Department: A Multi-Centre Derivation and Validation Study. Crit. Care Lond. Engl..

[31] Malik P., Patel U., Mehta D., Patel N., Kelkar R., Akrmah M., Gabrilove J.L., Sacks H. (2021). Biomarkers and Outcomes of COVID-19 Hospitalisations: Systematic Review and Meta-Analysis. BMJ Evid.-Based Med..

[32] Mohebbi A., Haybar H., Nakhaei Moghaddam F., Rasti Z., Vahid M.A., Saki N. (2023). Biomarkers of Endothelial Dysfunction Are Associated with Poor Outcome in COVID-19 Patients: A Systematic Review and Meta-Analysis. Rev. Med. Virol..

[33] Chenevier-Gobeaux C., Ducastel M., Meritet J.-F., Ballaa Y., Chapuis N., Pene F., Carlier N., Roche N., Szwebel T.-A., Terrier B. (2022). Plasma Endocan as a Biomarker of Thrombotic Events in COVID-19 Patients. J. Clin. Med..

[34] Görgün S., Cindoruk Ş., Özgen E., Yadigaroğlu M., Demir M.T., Yücel M., Akpınar Ç.K., Güzel M. (2021). Diagnostic and Prognostic Value of Serum Endocan Levels in Patients With COVID-19. Angiology.

[35] Pascreau T., Tcherakian C., Zuber B., Farfour E., Vasse M., Lassalle P. (2021). A High Blood Endocan Profile during COVID-19 Distinguishes Moderate from Severe Acute Respiratory Distress Syndrome. Crit. Care Lond. Engl..

[36] d’Alessandro M., Bergantini L., Cameli P., Curatola G., Remediani L., Sestini P., Bargagli E. (2021). Siena COVID Unit Peripheral Biomarkers’ Panel for Severe COVID-19 Patients. J. Med. Virol..

[37] Roedl K., Jarczak D., Fischer M., Haddad M., Boenisch O., de Heer G., Burdelski C., Frings D., Sensen B., Karakas M. (2021). MR-ProAdrenomedullin as a Predictor of Renal Replacement Therapy in a Cohort of Critically Ill Patients with COVID-19. Biomark. Biochem. Indic. Expo. Response Susceptibility Chem..

[38] Andrés C., Andaluz-Ojeda D., Cicuendez R., Nogales L., Martín S., Martin-Fernandez M., Almansa R., Calvo D., Esteban-Velasco M.C., Vaquero-Roncero L.M. (2020). MR- ProADM to Detect Specific Types of Organ Failure in Infection. Eur. J. Clin. Invest..

[39] Jewell P.D., Bramham K., Galloway J., Post F., Norton S., Teo J., Fisher R., Saha R., Hutchings S., Hopkins P. (2021). COVID-19-Related Acute Kidney Injury; Incidence, Risk Factors and Outcomes in a Large UK Cohort. BMC Nephrol..

