# Supplementary material for: MR-proADM as Prognostic Factor of Outcome in COVID-19 Patients
Source: Biomedicines. 2023 Jun 9;11(6):1680. doi: 10.3390/biomedicines11061680 (PMC10296224; doi:10.3390/biomedicines11061680)
Supplement: Supplementary file 1 [file biomedicines-11-01680-s001.zip › biomedicines-2422738-SI.pdf]

Supplementary Table

Table S1. Baseline demographic features, clinical and laboratory data of study population, stratified according to the availability of serum biomarkers

**Parameter**	**Only MR-proADM**	**MR-proADM + KL-6 + IL-6**	p-value
N°	52	22	
Age (years)	68.2 ± 16.8	64.6 ± 10.1	0.1071
Male gender (%)	32 (61.5%)	12 (54%)	0.6120
Medical history			
- Arterial hypertension (%)	32 (61.5%)	11 (50%)	0.4420
- Diabetes mellitus (%)	10 (19.2%)	5 (22.7%)	0.7578
- Atrial fibrillation (%)	7 (13.4%)	4 (18.1%)	0.7227
- Chronic kidney disease (%)	4 (7.6%)	1 (4.5%)	1
- Chronic obstructive pulmonary disease (%)	7 (13.4%)	2 (9%)	0.7163
- Active/previous cancer (%)	4 (7.6%)	2 (9%)	1
Clinical assessment			
- Hemoglobin (g/dl)	12.9 ± 2.04	13.6 ± 2.5	0.2558
- WBC ($x10^3$/mmc)	7.6 ± 4.5	7.3 ± 4.2	0.6652
- Lymphocytes ($x10^3$/mmc)	1.14 ± 0.9	0.9 ± 0.75	0.6263
- Neutrophils ($x10^3$/mmc)	6.1 ± 4.17	5.2 ± 4.6	0.4953
- Platelets ($x10^3$/mmc)	207.4 ± 91	228.9 ± 116.1	0.3867
- Creatinine (mg/dl)	1.2 ± 1	1.2 ± 0.8	0.2834
- CRP (mg/dl)	9.5 ± 23.2	10.2 ± 7.6	0.1065
- PCT (ng/ml)	0.7 ± 2.6	0.8 ± 4.6	0.6142
- INR	1.4 ± 1.1	1.3 ± 0.4	0.0952
- D-dimer (FEU/ml)	1223 ± 826	1443 ± 3268	0.7942
- Bilirubine (mg/dl)	0.9 ± 3.1	0.8 ± 2.5	0.5581
- LDH (U/l)	292.5 ± 115.4	335.6 ± 201	0.0526
- proBNP (pg/ml)	894 ± 1436	706 ± 2436	0.0879
- pH	6.9 ± 2.2	7.01 ± 1.6	0.1256
- paCO2 (mmHg)	34.1 ± 13.1	33.2 ± 12	0.6256
- paO2 (mmHg)	78.5 ± 36.2	82.6 ± 17.2	0.2556
- paO2/FiO2 (mmHg/%)	2.8 ± 1.3	2.9 ± 1.4	0.3694
- Serum lactate (mmol/l)	1.6 ± 0.7	1.2 ± 1	0.2114
